# Recent Advances in Progresses and Prospects of IL-37 in Central Nervous System Diseases

**DOI:** 10.3390/brainsci12060723

**Published:** 2022-05-31

**Authors:** Xinrui Li, Bing Yan, Jin Du, Shanshan Xu, Lu Liu, Caifei Pan, Xianhui Kang, Shengmei Zhu

**Affiliations:** 1 Department of Anesthesiology, The First Affiliated Hospital, Zhejiang University School of Medicine, Hangzhou 310003, China; 22118644@zju.edu.cn (X.L.); du4532380@163.com (J.D.); shanshan_xu2012@163.com (S.X.); liulu124@zju.edu.cn (L.L.); again_16@sina.com (C.P.); 2 Department of Anesthesiology, Haining People’s Hospital, Haining 314499, China; hnphyb@163.com; 3 China Coast Guard Hospital of the People‘s Armed Police Force, Jiaxing 314000, China

**Keywords:** Interleukin-37, central nervous system diseases, inflammatory, anti-inflammatory, cytokines

## Abstract

Interleukin-37 (IL-37) is an effective anti-inflammatory factor and acts through intracellular and extracellular pathways, inhibiting the effects of other inflammatory cytokines, such as IL-1β, IL-6, and tumor necrosis factor-α (TNF-α), thereby exerting powerful anti-inflammatory effects. In numerous recent studies, the anti-inflammatory effects of IL-37 have been described in many autoimmune diseases, colitis, and tumors. However, the current research on IL-37 in the field of the central nervous system (CNS) is not only less, but mainly for clinical research and little discussion of the mechanism. In this review, the role of IL-37 and its associated inflammatory factors in common CNS diseases are summarized, and their therapeutic potential in CNS diseases identified.

## 1. Introduction

Interleukin-37 (IL-37), a novel cytokine which was once considered as a member of IL-1 family, was reported to be a natural innate immune inhibitor [1]. Similar to other IL-1 family cytokines, IL-37 is encoded by chromosome 2 [2]. All members of this family share a common b-trefoil structure that includes 12 β-chains [3]. IL-37 is encoded by six exons. At present, five subtypes (IL-37a–e) formed by alternative splicing have been found, among which IL-37b has the largest molecular weight and is the most researched [4]. IL-37b contains five exons except exon 3, and IL-37c and IL-37e are predicted to be non-functional proteins because they lack one or more exons [5]. Based on the fact that most of the current research is on the IL-37b subtype, the IL-37 mentioned below in this article refers to IL-37b. In addition, IL-37a is the only form found in the brain. An unstable mRNA motif exists in exon 5. IL-37a is generally considered to be the functional subtype, but little research has been concentrated on this subtype alone [4]. Although IL-37 is widely expressed in many cell tissues in the human body, the concentration in the blood is extremely low (100 pg/mL) [6]. In some diseases involving inflammation, IL-37 is elevated due to inflammatory stimulation [7].

Previous research has shown the function of IL-37 in autoimmune diseases, including systemic lupus erythematosus [8], colitis [9], sepsis [10], asthma [1,11], and cancer [1,10,12]. However, the function and potential mechanisms of IL-37 in central nervous system (CNS) diseases have been investigated in only a few reviews. In the present review, current progress regarding IL-37 in CNS diseases is summarized and its therapeutic potential for CNS diseases identified.

## 2. About IL-37

### 2.1. Function

IL-37 was found in 2000 by several groups independently [11]; a homologue has not yet been identified in a mouse [4,7]. IL-37, originally named IL-1F7 [13], is mainly produced by Toll-like receptor (TLR)-activated macrophages [14]. The precursor of IL-37 (pre-IL-37) is cleaved by caspase-1 into mature IL-37, of which approximately 20% enters the nucleus, with the remaining released out of the cell with pre-IL-37 [15]. Many cells in the human body, including epithelial cells, keratinocytes, renal tubular epithelial cells, monocytes, activated B cells, plasma cells, dendritic cells (DCs), macrophages, and CD4+ Tregs, express IL-37 [5,7,16,17,18,19]. Reportedly, IL-37 expression is low in unstimulated peripheral blood mononuclear cells (PBMCs) and M1 macrophages, and IL-37 expression significantly increases after being activated by lipopolysaccharide (LPS) [20,21]. Human IL-37 precursor undergoes alternative splicing to form five different subtypes, and the IL-37b subtype has been the focus in most of the current studies [4]. IL-37 protein exists at low levels in human PBMCs and can be upregulated by inflammatory stimuli and cytokines, such as IL-1, IL-18, tumor necrosis factor (TNF), interferons (IFNs), and transforming growth factor (TGF) [7]. In addition, IL-37 is downregulated by IL-4, IL-12, IL-32, and granulocyte macrophage colony-stimulating factor [6,12].

IL-37 is constitutively expressed at low levels in various tissues, including lymph nodes, thymus, bone marrow, brain, intestines, airways, adipose, thymus, placenta, uterus, testis, heart, kidney, bone marrow, prostate, and breast [5,7,12,17,18,19]. IL-37 has a protective effect in a variety of diseases. Hui-min Chen et al. reported that compared with wild-type (WT) mice, IL-37 expression in DCs attenuates the ability of DCs to initiate contact hypersensitivity (CHS) responses in mice transgenic with human IL-37 (IL-37tg), demonstrating that IL-37 may be an immune tolerance factor [16]. Dov B. Ballak et al. revealed that IL-37 is expressed in human adipose tissue, and IL-37 can reduce diet-induced obesity in IL-37tg mice. In addition, IL-37 ameliorated diet-induced insulin resistance and improved insulin sensitivity in IL-37tg mice compared with WT mice, indicating potential as a treatment for obesity and type 2 diabetes [22]. Jilin Li et al. reported that IL-37 expression in an old endotoxemic mouse model suppressed myocardial inflammation-associated endotoxemia and improved left ventricle (LV) function, indicating its protective function in septic myocarditis [23]. Tianheng Hou et al. proved that IL-37 could reduce Der p1-induced thymic stromal lymphopoietin (TSLP) overexpression in HaCa T cells, and decreased TSLP receptors and basophil activation marker CD203c in vitro. In vivo experiments in an atopic dermatitis mouse model showed alternative depletion of basophils rescued atopic dermatitis symptoms and significantly lowered the helper T cell 2 (Th2) and eosinophil populations in the ear and spleen [24]. In other studies, therapeutic effects of IL-37 on allergic diseases, autoimmune diseases, and other immune system diseases have been reported [6,25,26,27,28]. In addition, IL-37 reportedly exerts tumor-inhibiting effects in a variety of cancers, such as breast, cervical, melanoma, and non-small cell lung cancer [12,29]; refer to the relevant review for details.

### 2.2. Pathway

IL-37 exerts anti-inflammatory effects through intracellular and extracellular pathways [6]. In the intracellular pathway, the precursor IL-37 (pro-IL-37) is cleaved by caspase-1 to produce mature IL-37 after activation by lipopolysaccharides(LPS) [30,31]. A possible cleavage site for caspase-1 is located in exon 1 between the D20 and E21 residues of IL-37 [32]. However, Ana-Maria Bulau et al. previously demonstrated that caspase-1 inhibitors only partially inhibit the processing of IL-37, indicating that caspase-1 is not the only enzyme responsible for the processing of IL-37 [30]. Human embryonic kidney 293 (HEK 293) or Chinese hamster ovary (CHO) cells transfected with IL-37 precursor release IL-37 from amino acid V46, indicating there is a second cleavage site in the sequence encoded in exon 2 [33]. In addition to caspase-1, caspase-4 was shown to cleave pro-IL-37 to a certain extent [32]. IL-37 binds with drosophila mothers against decapentaplegic protein 3 (Smad3) to form a complex in the cytoplasm; then, the complex enters the nucleus to regulate the transcription process, such as nuclear factor-κB (NF-κB) and mitogen-activated protein kinase (MAPK) pathways, thereby inhibiting the transcription process of some inflammatory cytokines [6,12,34].

**Figure 1:**
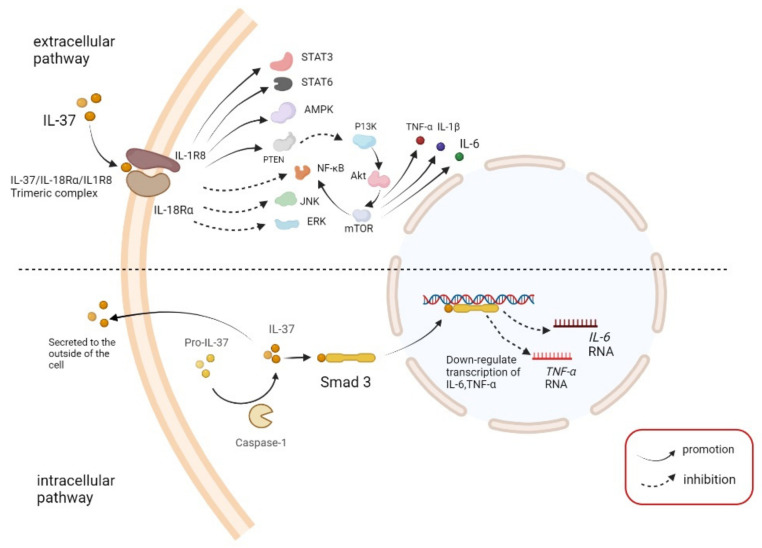
Pathway through which Interleukin-37 (IL-37) exerts anti-inflammatory effects. In extracellular pathway, IL-37 binds with IL-18 receptor α chain (IL-18Rα) and IL-1 receptor 8 (IL-1R8) to form a trimeric complex. The trimeric complex activates adenosine 5′-monophosphate-activated protein kinase (AMPK), signal transducer and activator of transcription 3 (STAT3), STAT6 and phosphate and tension homology deleted on chromosome ten (PTEN), and inhibits the pathway of c-Jun N-terminal kinase (JNK), extracellular regulated protein kinases (ERK) and nuclear factor-κB (NF-κB). At the same time, PTEN inhibits the PI3K/AKT/mTOR pathway, thereby inhibiting the production of NF-κB and pro-inflammatory cytokines, including IL-6, tumor necrosis factor-α (TNF-α) and IL-1β. In intracellular pathway, pro-IL-37 is cleaved by caspase-1 to become IL-37. A part of IL-37 is released outside the cell, and a part binds to drosophila mothers against decapentaplegic 3 (Smad3) in the cytoplasm. After entering the nucleus, it inhibits the transcription of other inflammatory cytokines, such as IL-6 and TNF-α.

In the extracellular pathway, IL-37 binds to IL-18 receptor α chain (IL-18Rα) to exert an anti-inflammatory effect [35]. IL-37 binds to IL-18Rα to recruit IL-1 receptor 8 (IL-1R8, also named single Ig IL-1R-related molecule, SIGIRR), to form a trimeric complex (IL-37/IL-18Rα/IL-1R8) [36]. When adenosine 5′-monophosphate-activated protein kinase (AMPK) is increased, signal transducer and activator of transcription 3 (STAT3), STAT6, phosphatase and tensin homologs, and other factors inhibit the inflammatory response induced by IL-18 and downregulate the expression of IFN-γ and transcription factor NF-κB [37]. SIGIRR is the only receptor containing a TLR domain with a single immunoglobulin domain. Although SIGIRR has an immunoglobulin domain, it cannot bind to IL-1 or enhance IL-1-dependent signaling. SIGIRR is a negative regulator of the inflammatory response and inhibits the inflammation process of IL-1 and IL-18 [38]. Both pro-IL-37 and mature IL-37 can bind to IL18Rα but the binding of the mature form is approximately 5–10-fold stronger than the immature form [32]. IL-IR8 is necessary for activation of the anti-inflammatory signal transducer and transcriptional activator STAT3 in splenic DCs and macrophages [36]. However, IL-37 is not a receptor antagonist of IL-18Rα [39]. SIGIRR was shown unstable in response to IL-37, and IL-37 can mediate the ubiquitination and degradation of SIGIRR through glycogen synthesis kinase 3β (GSK3β) [40]. See Figure 1 for a summary of the IL-37 pathway.

## 3. IL-37 in CNS Diseases

### 3.1. Acute Spinal Cord Injury (ASCI)

Acute spinal cord injury (ASCI) is common in serious trauma caused by transport accidents, low-height falls, and other accidents [41]. The quality of life of ASCI patients is reduced, the prognosis very poor, and the mortality rate is high [42]. According to survey results, the mortality rate of individuals over 65 years of age suffering from SCI is 36.5% [43]. Therefore, improving the prognosis and survival rate of ASCI patients is important.

Marina Coll-Miró et al. transferred the human IL-37 gene into mice to produce hIL-37tg mice. The WT and hIL-37tg mice were subjected to spinal cord contusion injury. The authors found that compared with WT mice, hIL-37tg mice had more myelin and neurons preserved, and maintained a lower level of cytokines and chemokines (e.g., an 80% reduction in IL-6). The authors infused recombinant human IL-37 (rIL-37) into the lesion site via a glass capillary 5 min after the contusion injury, and found the mice injected with rIL-37 had a greater extensive movement of the ankle restored and increased their speed on a treadmill by 50% [44]. An experiment conducted on 148 patients showed that serum IL-37 levels were significantly higher within 24 h of ASCI compared with the control group (*p* < 0.05). Serum IL-37 concentration in patients with SCI is negatively correlated with American Spinal Cord Injury Association (ASIA) exercise score (*p* < 0.05) [45]. The above results indicate IL-37 may be a potential therapeutic target and a biomarker after ASCI. IL-37 may inhibit the inflammatory response after ASCI to produce neurological protection and recovery. Jesus Amo-Aparicio et al. used a hIL-37D20ATg transgenic mouse model lacking the IL-37 intracellular pathway to prove that the neuroprotection role of IL-37 after SCI does not rely on the intracellular pathway rather than the extracellular pathway. Their study demonstrated IL-37 exerts an anti-inflammatory effect by binding IL-1R8 [46].

### 3.2. Demyelinating Disease

Multiple sclerosis (MS) is a chronic, predominantly immune-mediated disease of the brain and spinal cord, and a common cause of neurological disability in young adults, affecting more than 2.5 million individuals globally [47,48]. Alba Sánchez-Fernández et al. used the experimental autoimmune encephalomyelitis (EAE), a murine model of MS, hypothesizing that IL-37 reduces inflammation and protects against neurological deficits and myelin loss in EAE mice by combining with IL-1R5/IL-1R8. The authors found that transgenic expression of IL-37 reduces neurological deficits and inflammation in the spinal cord of EAE mice. However, in the transgenic homozygote of human IL-37 (hIL-37tg) mice lacking IL-1R8, the beneficial effects of IL-37 were completely absent in demyelinating disease of the CNS, indicating IL-37 acts with IL-1R8 [49].

In another study, IL-37 level in a cluster of differentiating CD4+ T cells from MS patients was decreased in vitro compared with healthy controls based on in silico analysis [50]. IL-37 expression was observed in PBMCs from MS patients during the exacerbation of the disease [50]. In addition, higher IL-37 levels showed an inhibitory effect on MS recurrence; however, obvious effects were not observed with IL-1R8 and IL-18R1. Higher IL-37 levels were associated with younger age and lower Multiple Sclerosis Severity Score (MSSS) [50]. After testing serum IL-37 levels in 84 MS patients and 75 healthy controls, Ebrahim Kouchaki et al. found IL-37 levels in MS patients were higher than in the control group (*p* < 0.001) [51]. The research by Farrokhi. M et al. of 122 MS patients and 49 healthy subjects showed the IL-37 levels were higher in the MS patients than in the controls [52].

Guillain-Barré syndrome (GBS) is the most common and severe acute paralytic neuropathy. Globally, approximately 100,000 people develop this disorder annually [53]. GBS is considered an immune-mediated disease, possibly triggered by a recent infection, and driven by an immune attack targeting the peripheral nervous system [54]. Approximately 25% of patients have respiratory insufficiency and many patients show signs of autonomic dysfunction [55]. GBS is the most common cause of acute flaccid paralysis, which leads to disability and high risk of mortality. The specific pathogenic mechanism of GBS remains unclear. Some patients have been reported to have an infectious disease before the onset of the disease; thus, the disease is hypothetically an immune-mediated disease [54]. Cong Li et al. measured the IL-37 levels in the cerebrospinal fluid (CF) and plasma of 25 GBS patients and 20 healthy controls and found the IL-37 levels in the CF and plasma of GBS patients were significantly higher than in the healthy controls (*p* = 0.0002 and *p* < 0.0001) [56]. This result indicates that during the pathogenesis of GBS, pro-inflammatory cytokines may promote the expression of anti-inflammatory IL-37, thereby downregulating the excessive inflammatory response, similar to the results of several previous studies [9,57,58].

### 3.3. Alzheimer’s Disease (AD)

Alzheimer’s disease (AD) is a neurodegenerative disease highly correlated with age [59]. From 1999 to 2018, the number of deaths from AD in the United States increased [60]. Currently, an estimated 6.2 million Americans 65 years of age and older have AD, and if no effective treatment is found, this number will continue to increase [61]. In many current studies, the relationship between inflammation and AD is being investigated [62,63]. Aging tissue cells secrete inflammation and immune-related cytokines, such as interleukins, chemokines, growth factors, and proteases, which constitute senescence-associated secretory phenotype (SASP) [64]. Astrocytes and microglia surround neuropathic plaques composed of amyloid β-protein (Aβ) and neurofibrillary tangles [65]. Microglia release cytokines and cause neuroinflammation [66]. Among them, M1 type microglia, which are activated by LPS, IFN-γ or TNF-α, secrete classic inflammatory cytokines, such as IL-1β, TNF-α, STAT3, IL-6, IL-12, and IL-23, and free radicals such as reactive oxygen species (ROS) [67]. Another M2 anti-inflammatory phenotype promotes tissue remodeling by releasing high levels of anti-inflammatory cytokines, such as IL-10, IL-4, IL-13 and transforming growth factor-β, and low levels of pro-inflammatory cytokines/repair and angiogenesis [67].

In previous studies, a high-fat diet was shown to induce insulin resistance, reduce the transport of glucose into the brain, and ultimately lead to neuronal stress (elevated neuronal corticosterone) [68]. The intake of fructose promotes the synthesis of triglycerides, gluconeogenesis and insulin resistance, and ultimately accelerates the progression of AD [69]. A rat model of AD was established by Mohamed, R.A. et al. with a synergistic high fat/high fructose diet (HFFH) and LPS injection. The authors found the hippocampal AD marker Aβ1-42 and the inflammatory marker IL-1β in the mouse model were increased 3.2-fold and 5.6-fold, respectively, compared with the controls [70]. The combined use of palonosetron and methyllycaconitine (MLA) restored the activity of caspase-1 protein in AD rats and reduced the reactivity of astrocytes [70].

The role of many cytokines in AD has been elucidated [71]. Tau and Aβ modified by advanced glycation end products stimulate human neurons to produce IL-6 [72]. IL-6 can also activate janus kinase (JAK)/STATs, N-methyl-D-aspartate (NMDA) receptor, and mitogen-activated protein kinase (MAPK)-p38, which are involved in the hyperphosphorylation of tau [72]. However, in an in vivo study, IL-6 overexpression induced the reduction in neuroinflammation at the Aβ level rather than aggravating the pathology of Aβ plaques [73]. Irina Belaya et al. reported regular exercise can modulate iron homeostasis in WT mice and in a mouse model of AD via the IL-6/STAT3/JAK1 pathway [74], indicating that M2 type microglia are mainly activated, which enhances the phagocytic function of Aβ [73]. The combination of IL-18 and its receptor complex can activate c-Jun N-terminal kinase (JNK) and MAPK-p38, thereby activating endogenous and exogenous pro-apoptotic signaling pathways [75]. This effect may be achieved by inducing the expression of p53 and Fas ligand, indicating IL-18 can promote the progression of AD [75,76]. However, research on the role of IL-37 in AD is limited. Exploring the roles of IL-37 in AD can be a promising direction for future research because caspase-1 is also an enzyme that cleaves the precursor of IL-37 and renders it active [31]. In addition, IL-37 has been shown to inhibit the inflammatory effects of other inflammatory cytokines, such as IL-6 and IL-1β in many other diseases [77,78,79,80,81,82]. In a temporomandibular joint study, IL-37 could convert M1 type macrophages to M2 type, thus alleviating inflammation [83]. Therefore, future research should focus on the protective effect of IL-37 on the progression of AD.

### 3.4. Stroke

Stroke is the second leading cause of death and disability in the world, a non-communicable disease that seriously endangers the health of Chinese people [84]. In an epidemiological survey of 10,926 participants, 602 cases were diagnosed as ischemic stroke (IS), 151 cases as hemorrhagic stroke, and 22 cases as both hemorrhage and IS. The crude prevalence rates of total stroke, IS, and hemorrhagic stroke were 6690.5/100,000, 5509.8/100,000, and 1382.0/100,000, respectively, and the standardized rates were 4903.8/100,000, 4041.7/100,000, and 990.9/100,000, respectively [85].

Feng Zhang et al. measured the serum IL-37 levels using enzyme-linked immunosorbent assay in 152 patients admitted to the hospital due to acute IS, and in 45 healthy controls. The authors found serum IL-37 levels in IS patients were significantly higher than in controls (182.26 vs. 97.89 pg/mL, *p* < 0.001) and associated with the National Institutes of Health Stroke Scale (NIHSS) scores (r = 0.521, *p* < 0.0001) and lesion volume (r = 0.442, *p* < 0.0001). Notably, elevated plasma IL-37 levels were independently associated with unfavorable 3-month outcomes (adjusted odds ratio = 1.033, *p* = 0.001, 95% confidence interval, CI:1.015–1.056) [86]. In another study, the serum IL-37 levels were measured in 310 IS patients who were followed up for 3 months to determine the relationship between serum IL-37 and recurrence of IS. The study results showed the median IL-37 serum level in the IS patients was 344.1 pg/mL (interquartile range, IQR, 284.4–405.3) and 122.3 pg/mL (IQR, 104.4–1444.0) in the controls, which was significantly lower. The size of the lesion area observed on magnetic resonance imaging (MRI) positively correlated with serum IL-37 levels. In that study, 36 patients experienced relapse of IS within 3 months, and their serum IL-37 levels were higher than in patients who did not relapse (417.0 pg/mL; IQR, 359.3–436.1 vs. 333.3 pg/mL; IQR, 279.0–391.0), indicating IL-37 levels are associated with the recurrence of IS. Based on receiver operating characteristic (ROC) analysis, the authors determined the IL-37 level cut-off value to diagnose IS was 193.0 pg/mL, the cut-off value to diagnose moderate-to-high clinical severity (NIHSS score > 5) was 374.0 pg/mL, and the cut-off value to predict recurrence was 406.8 pg/mL [87].

To date, research on the relationship between IL-37 and hemorrhagic stroke is scarce. However, an increase in the white blood cell/lymphocyte ratio in patients with IS was positively correlated with the probability of hemorrhagic transformation [88], indicating hemorrhagic transformation, a serious complication of severe IS, may be associated with inflammation possibly related to the destruction of the blood–brain barrier by neutrophils [89,90].

## 4. Conclusions

IL-37 is a potent endogenous anti-inflammatory factor of the IL-1 family. IL-37 suppresses other inflammatory cytokines, thus inhibiting the progression of disease [3]. In an in vitro experiment, siRNA to IL-37 (siIL-37) was transfected into human PBMCs stimulated by LPS. After IL-37 expression was specifically silenced, the production of IL-6 and other cytokines increased in a dose-dependent manner [82], indicating IL-37 can inhibit the inflammatory effect of IL-6 and play an anti-inflammatory role. Irene Tsilioni et al. found that neuropeptide NT can stimulate human microglia to secrete IL-1β, CXCL8, and other cytokines that can be inhibited by IL-37 [91]; however, the specific mechanism underlying this inhibition remains unclear. In 293T cells, overexpression of IL-37 restored the viability of cells damaged by homocysteine and reduced the release of lactate dehydrogenase, pro-inflammatory cytokines IL-1β, IL-6, and TNF-α [92]. In liver cancer cells, IL-37 inhibited IL-6 expression by hindering the STAT3 pathway, thereby inhibiting the inflammatory response of IL-6 [10]. IL-37 inhibited the expression and phosphorylation of STAT3, thus hindering the inflammatory effects of TNF-γ and IL-1β mediated by STAT3 [93].

IL-18, a member of the IL-1 family, was first discovered in 1989 and is an inflammatory factor [35]. IL-18 is the main inducer of IFN-γ, playing an important role in promoting the activation of inflammatory helper T cell 1 (Th1) and natural killer (NK) cells [94]. The IL-37/IL-18Rα complex combines with IL-1R8 to promote anti-inflammatory effects by activating STAT3 and transmitting inhibitory signals [7,32,36,39]. IL-37 is an endogenous factor that inhibits IL-18 effects. IL-37 has high homology with IL-18 and IL-18BP binds to IL-37. IL-18BP is a structural secreted protein with a high affinity for IL-18 [37,95], which when combined with IL-37 can enhance the ability of IL-18BP to inhibit IL-18-stimulated ITF-γ induction and inflammation [7,35,37,39]. However, Suzhao Li et al. reported that at micromolar concentrations, IL-37 binds to IL-18Rα and recruits IL-1R8, which may result in anti-inflammatory effects. At picomolar concentrations, the IL-37/IL-18Rα complex may recruit IL-18Rβ and the corresponding IL-18 signal, which may be associated with the inflammation process [21].

Based on the above studies, IL-37 has significant therapeutic potential through suppressing inflammation by inhibiting the transcription and expression of other inflammatory factors, including IL-6, IL-1β, IL-18 [6,12,34]. In tumors and some autoimmune diseases, IL-37 has exhibited its powerful anti-inflammatory ability [12]. Based on that, many CNS diseases are closely related to inflammation, and future research on CNS diseases may focus on the anti-inflammation function in some diseases, as well as expound and reveal its anti-inflammatory effect and mechanism. Due to the lack of research on the a subtype, and as IL-37a is the only subtype expressed in the brain, future research may focus on the protective effect of this subtype on the brain and explore the therapeutic and protective effects.
